# R Version of the Kedem–Katchalsky–Peusner Equations for Liquid Interface Potentials in a Membrane System

**DOI:** 10.3390/e27020169

**Published:** 2025-02-06

**Authors:** Andrzej Ślęzak, Sławomir M. Grzegorczyn

**Affiliations:** 1 Department of Health Sciences and Physiotherapy, Collegium Medicum, Jan Dlugosz University, 13/15 Armia Krajowa Al, 42200 Częstochowa, Poland; 2 Department of Biophysics, Faculty of Medical Sciences in Zabrze, Medical University of Silesia, 19 H. Jordan Str., 41808 Zabrze, Poland

**Keywords:** membrane transport, R version of the Kedem–Katchalsky–Peusner equations, Peusner coefficients, internal energy conversion, S entropy

## Abstract

Peusner’s network thermodynamics (PNT) is an important way of describing processes in nonequilibrium thermodynamics. PNT allows energy transport and conversion processes in membrane systems to be described. This conversion concerns internal energy transformation into free and dissipated energies linked with the membrane transport of solutes. A transformation of the Kedem–Katchalsky (K-K) equations into the R variant of Kedem–Katchalsky–Peusner (K-K-P) equations was developed for the transport of binary electrolytic solutions through a membrane. The procedure was verified for a system in which a membrane Ultra Flo 145 Dialyser separated aqueous NaCl solutions. Peusner coefficients were calculated by the transformation of the K-K coefficients. Next, the coupling coefficients of the membrane processes and energy fluxes for electrolyte solutions transported through the membrane were calculated based on the Peusner coefficients. The efficiency of energy conversion in the membrane transport processes was estimated, and this coefficient increased nonlinearly with the increase in the solute concentration in the membrane. In addition, the energy fluxes as functions of ionic current density for constant solute fluxes were also investigated for membrane transport processes in the Ultra Flo 145 Dialyser membrane.

## 1. Introduction

Membrane transport, described by nonequilibrium thermodynamics, includes processes occurring in various types of biological or artificial membrane systems of different structures, selectivity, and, therefore, function [1]. The investigation of membrane transport processes provides important information on cognitive and applied characteristics for many areas of biomedical or industrial technologies. The typical examples of these applications are wastewater and water treatment systems, fuel cells, membrane dressings supporting the healing of chronic wounds, controlled drug release systems, or bioreactors, allowing the strategies to combat bacterial infections by means of lytic phages in combination with known and new antimicrobial agents to be examined [2,3,4]. The selective barrier in such systems is the suitable membrane, ensuring functionality and efficiency. Such membranes include, among others, cellulose acetate or bacterial cellulose membranes and polymeric membranes of different structures and compositions made of polyvinyl chloride [5,6].

One of the physical quantities which characterise nonequilibrium systems is thermodynamic entropy (S-entropy) [7]. This parameter describes the degree of irreversibility of physicochemical processes that fulfil the second law of thermodynamics.This means that entropy is a measure of dissipated energy for irreversible processes of mass, charge, energy, and momentum transport in physicochemical systems, such as membranes, among others. In turn, the value of dissipated energy is equal to the product of entropy production and absolute temperature. The function of energy dissipation is the basis for deriving the membrane transport equations, as well as the internal energy conversion equation [8].

The Kedem–Katchalsky equations [9], which describe the membrane transport, appeared in science in the late 1950s and found applications in many areas of science, technology, and biomedicine. In recent years, new research tools have been developed within the framework of network thermodynamics (NT). The first principles of NT appeared in the 1960s with the idea of the Paynter bond graphs method [10] and with the relationship between irreversible transport systems and electrical networks described by Meixner [11]. In the early 1970s, articles by Peusner [12] and Oster and Perelson and Katchalsky [13] were published and constitute the pillars of the NT. Currently, NT is a synthesis of classical nonequilibrium thermodynamics, differential geometry, graphs, and electrical circuit theory [14,15,16].

Practical applications of membrane transport analysis are based on Peusner’s ideas of NT regarding the use of nonequilibrium thermodynamics and the symbolism of the analogue electrical circuit theory (Kirchhoff’s current and voltage laws, Tellegen’s principle, etc.) [14,15,16,17,18] and the ideas of Oster, Perelson, and Katchalsky [13] based on the use of Paynter’s graph method [1]. Currently, there are two branches of network thermodynamics: the network thermodynamics of Peusner (Peusner NT) and the network thermodynamics of Oster, Perelson, and Katchalsky (Oster, Perelson, Katchalsky NT). In “his” thermodynamics, Leonardo Peusner focused on the mathematical and formal aspects of network thermodynamics, introducing, among other things, symmetric ($L_{ij}$, $R_{ij}$) and hybrid ($H_{ij}$, $P_{ij}$) parameters now called symmetric and hybrid Peusner coefficients, respectively, generalising network transformations and developing the matrix formalism. The parameters $L_{ij}$, $R_{ij}$, $H_{ij},$ and $P_{ij}$ allow the creation of general relationships in which thermodynamic flux can be a function of both forces and other fluxes, which better reflects the actual coupling between processes. His work provided the basis for a general and accurate tool for modelling nonequilibrium systems. In Peusner’s NT, a system is represented by a network of elements (resistors, transformers, and sources) connected by nodes. Network transformations allow this network to be manipulated without changing its physical properties, that is, without changing the relationship between thermodynamic fluxes and forces. Peusner NT methods allow for the simplification of complex mathematical models, the derivation of equations, and the analysis of their sensitivity. Understanding these transformations is crucial for the effective use of Peusner NT in membrane transport research.

Oster, Perelson, and Katchalsky (after Aharon Katchalsky’s death in 1971) Oster and Perelson focused on the applications of network thermodynamics in biology, showing how it can be used to model specific biological processes and developing methodologies to facilitate these applications. George Oster was intensely active in research until his death in 2018. Both Peusner’s NT and the Oster, Perelson, and Katchalsky NT offer tools for analysing and modelling complex physicochemical biological and medical processes [19,20,21,22,23,24,25,26,27,28]. Their ability to take into account coupling and flexibility in modelling different types of systems makes it a promising approach for studying the functioning of living organisms, developing new tools for analysing and modelling nonequilibrium processes, including membrane transport, and developing new methods for medical diagnostics and therapy. These include modelling drug transport across cell membranes, ion flow through ion channels and ion pumps; water flow through biological membranes; the optimisation of dialysis therapies; modelling interactions between genes and proteins; and complex networks of metabolic reactions and metabolite transport.

**Figure 1:**
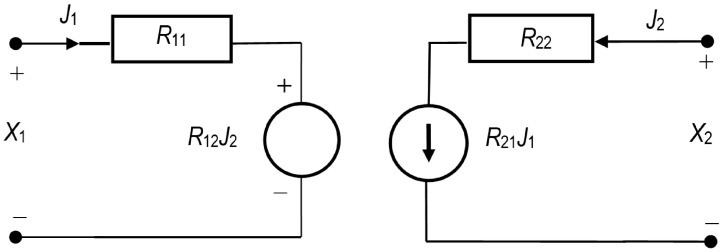
R representation of the phenomenological equations. Thermodynamic forces ($X_{1},\;X_{2}$) controlling the sources of fluxes ($J_{1},\;J_{2}$) are placed in parallel with resistances ($R_{11}$, $R_{22}$) [14,16].

The labels *L*, *R*, *H*, and *P* of transducers are in accordance with Peusner’s idea and the electrical circuit theory [14,15,16]. Each transducer is composed of two dissipative elements (conductance or resistance) and two controlled sources (force or flow). In this paper, the version of equations containing the *R* coefficients is considered. The *R* representation of phenomenological equations in which the force-controlled sources are placed in series with resistances is presented in Figure 1.

The ‘resistive’ formulation of the *R* version of phenomenological equations can be presented as follows:

(1) $$X_{i}=\sum_{k=1}^{n}R_{ik}J_{k}$$

The definitions of symbols in Equation (1) are the same as in the Onsager notation: $X_{i}$—thermodynamic force; $R_{ik}$—phenomenological resistance coefficient; and $J_{k}$—thermodynamic flux. $R_{ik}$ coefficients may or may not satisfy the Onsager symmetry relation for cross coefficients [17,29,30]. As mentioned earlier, NT presents this equation with linear resistances, sources of force, and flow so as to fulfil Kirchhoff’s laws. Equation (1) can be transformed, maintaining the coupled nature of thermodynamic forces and flows.

Previous papers have presented procedures for the analysis of the membrane transport of binary and ternary non-electrolyte solutions by means of the *R*-version of the K-K-P equations [17]. A procedure for the conversion of chemical energy to free energy was also presented [8]. The first step in this procedure is the calculation of dissipation energy by means of the *R*-version of the K-K-P equations. In addition, the hydrostatic and osmotic pressure differences and volume and solute fluxes were used in these procedures. Paper [8] presents a transformation of K-K equations for binary electrolyte solutions into K-K-P equations by applying a method elaborated on the basis of PNT. For aqueous electrolyte solutions with a concentration field and an electric field imposed on a membrane system, a method of conversion of internal energy into free energy was developed by means of the *L*-version of the K-K-P equations [8].

In the present paper, the *R*-version of the K-K-P equations for binary electrolyte solutions is presented. Using the *R*-version of the K-K-P equations, the conversion of internal energy to free and dissipated energy for aqueous electrolyte solutions with concentration gradients and an electric field in a membrane system is elaborated. In addition, the procedure for deriving the *R*-version of the K-K-P equations describing the membrane transport of homogeneous electrolyte solutions and the equations representing the *R*-version of the transport-resistant parameters $R_{ij}$ and the coupling coefficients between observed processes ($r_{ij}$) are presented. The energy conversion efficiency coefficient ((${e_{R})}_{max}$) and the equation for the S-energy dissipation function flux (${\left(\Phi_{S}\right)}_{R}$) derived from K-K-P formalism are also presented. To calculate the characteristics of fluxes of energy dissipation (${\left(\Phi_{S}\right)}_{R}$) and useful $\left({\left(\Phi_{F}\right)}_{R}\right)$ functions as functions of ionic current density for constant solute fluxes, the derived equations for resistant parameters and different solute concentrations in the membrane ($C_{s}$) were used for the Ultra Flo 145 Dialyzer membrane and aqueous NaCl solutions.

## 2. Materials and Methods

### 2.1. Membrane System

**Figure 2:**
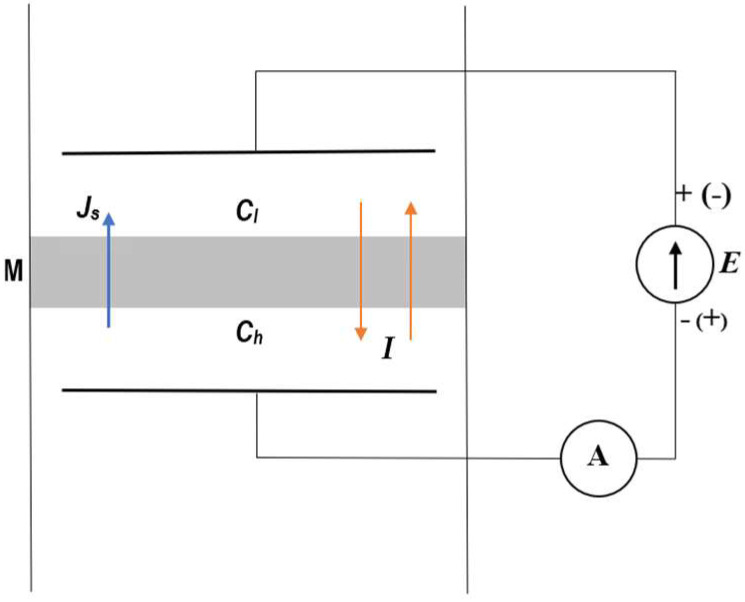
Scheme of the measuring system: M—membrane; $C_{h}$ and $C_{l}$—NaCl solution concentrations ($C_{h}$ > $C_{l}$); $J_{s}$—solute flux; I—electric ionic current; E—electrode potentials difference.

The measurement system is shown in Figure 2. This system is composed of a membrane (M), located in a horizontal plane and two aqueous NaCl solutions with concentrations at the initial moment: $C_{h}$ and $C_{l}$ = const. ($C_{h}$ ≥ $C_{l}$). The density of solutions with concentrations $C_{h}$ and $C_{l}$ fulfilled the following condition: $\rho_{h}$ ≥ $\rho_{l}=$ constant. In the measurement system, two thermodynamic forces $∆\pi =RT(C_{h}-C_{l})$ and $E=(E_{h}-E_{l}$) generating two fluxes ($J_{s}$, I) were used. Ag/AgCl electrodes were located in $C_{l}$ and $C_{h}$ solutions between which the voltage $E=E_{h}-E_{l}$ was applied.

### 2.2. R Version of the Kedem–Katchalsky–Peusner Equations

The R version of the Kedem–Katchalsky–Peusner equations for homogeneous electrolyte solutions is obtained by the appropriate transformation of the classical Kedem–Katchalsky equations with volume flux through the membrane equal to zero ($J_{v}$ = 0) [9]. Kedem–Katchalsky equations can be presented as follows:

(2) $$J_{s}={C_{s}\omega }_{s}\frac{{∆\pi }_{s}}{C_{s}}+\frac{\tau_{s}}{z_{s}F}I$$

(3) $$I=\frac{\kappa \tau_{s}}{z_{s}F}\frac{{∆\pi }_{s}}{C_{s}}+\kappa E$$

where $\omega_{s}$—coefficient of diffusion permeability; RT—the product of the gas constant and the absolute temperature; ∆C = $C_{h}-C_{l}$ ($C_{h}>C_{l}$)—difference in concentrations on the membrane; $C_{s}=(C_{h}-C_{l}){\left(lnC_{h}{C_{l}}^{-1}\right)}^{-1}$ = $∆\pi_{s}{\left(RTlnC_{h}{C_{l}}^{-1}\right)}^{-1}$ ≈ 0.5${(C}_{h}+C_{l})$—average concentration of the solution in the membrane; $\tau_{s}$—transference number; F—Faraday constant; κ—conductance coefficient; $z_{s}$—valence of *s* ion; main thermodynamic fluxes: $J_{s}$—solute flux; *I*—electric ionic current density; main thermodynamic forces: $∆\pi_{s}$ = RT∆C—osmotic pressure difference; and E—the potential difference (voltage) through membrane. The phenomenological coefficients appearing in Equations (2) and (3) are defined by the following expressions: $\omega_{s}={\left(\frac{J_{s}}{{∆\pi }_{s}}\right)}_{\;I=0}$, $\kappa ={\left(\frac{I}{E}\right)}_{\;{∆\pi }_{s}=RT\left(C_{h}-C_{l}\right)=0,\;{\;C}_{h}=C_{l}\neq 0}$, and $\tau_{s}=z_{s}F{\left(\frac{J_{s}}{I}\right)}_{{∆\pi }_{s}=RT\left(C_{h}-C_{l}\right)=0,\;{\;C}_{h}=C_{l}\neq 0\;}$.

Transforming Equations (2) and (3) by means of PNT methods, we obtain the R version of these equations:

(4) $$\left(\begin{array}{c} \frac{{∆\pi }_{s}}{C_{s}}\\ E \end{array}\right)=R\cdot \left(\begin{array}{c} J_{s}\\ I \end{array}\right)$$

where

(5) $$R=\left(\begin{array}{cc} \frac{1}{{C_{s}\omega }_{s}} & -\frac{\tau_{s}\kappa }{z_{s}F{C_{s}\omega }_{s}}\\ -\frac{\tau_{s}\kappa }{z_{s}F{C_{s}\omega }_{s}} & \frac{1}{\kappa }+\frac{{\tau_{s}}^{2}}{{z_{s}}^{2}F^{2}{C_{s}\omega }_{s}} \end{array}\right)$$

Equation (5) is one of the R forms of the K-K equations obtained by the symmetrical transformation of PNT.

**Figure 3:**
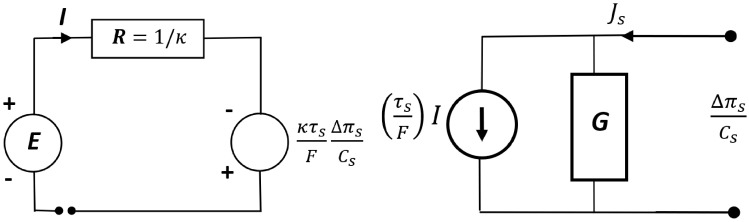
Electrical circuit diagrams of the phenomenological liquid junction potential equations with $G=\omega_{s}C_{s}$ [15].

The comparison of non-diagonal coefficients of the matrix *R* (5) shows that $R_{12}$ = $R_{21}$. For the fluxes $J_{s}$ and I coupled with the forces ${∆\pi }_{s}/C_{s}$ and E, the relations $R_{11}R_{22}$ ≥ ${R_{12}}^{2}$ and $R_{11}R_{22}$ ≥ ${R_{21}}^{2}$ are valid. Furthermore, the flux $J_{s}$ can only be coupled to the force E if $R_{12}$ ≠ 0. In turn, the flux I can only be coupled to the force ${∆\pi }_{s}/C_{s}$ if $R_{21}$ ≠ 0. The schematic electrical diagrams for Equations (2) and (3) and NaCl solutions are presented in Figure 3.

Resistant non-diagonal coefficients $R_{ij(i\neq j)}$ describe the connections between different irreversible processes. The coefficient $r_{12}$ in Equation (6) was constructed from the coefficients $R_{ij}$ (*i*, *j* ∈ {1, 2})

(6) $$r_{12}=\frac{{-R}_{12}}{\sqrt{R_{11}R_{22}}}={\left(\frac{{\kappa \tau_{s}}^{2}}{C_{s}\omega_{s}{{z_{s}}^{2}F}^{2}+{\tau_{s}}^{2}\kappa }\right)}^{\frac{1}{2}}$$

and determines the degree of coupling between observed processes (Kedem and Caplan coefficient) [31,32]. If $r_{12}$ = 0, irreversible processes are independent, while when $r_{12}$ = ±1, irreversible processes are maximally coupled.

The concept of the degree of coupling was used to determine the energy conversion efficiency. The maximum value of this coefficient is determined by the following expression:

(7) $${\left(e_{R}\right)}_{max}=\frac{R_{12}R_{21}}{R_{11}R_{22}{\left(1+\sqrt{1-\frac{R_{12}R_{21}}{R_{11}R_{22}}}\right)}^{2}}$$

Equation (7) illustrates the relationship between the degree of coupling and the maximum efficiency of energy conversion. Full coupling ($r_{12}$ = 1) occurs at $({e_{r})}_{max}\;$ = 1. This means that the stationary states of flow characterised by minimum entropy production are identical to the state with maximum efficiency. This parameter determines the efficiency of physicochemical and biological energy conversion systems.

### 2.3. Mathematical Model of Energy Conversion in the Membrane System

The measure of S-energy dissipation is the so-called dissipation function $\Phi_{S}$, which is equal to the product of absolute temperature (*T*) and S-entropy production ($d_{i}S/dt$). Using the procedure described in the previous paper [8], the mathematical expressions for *S*-energy dissipation in a system with a membrane and two homogeneous electrolytic solutions of different concentrations were derived.

For the stationary membrane transport of homogeneous binary electrolytic solutions and $J_{v}$ = 0, the equation for the R version of the dissipation function took the following form:

(8) $${\left(\Phi_{S}\right)}_{R}={\left(\Phi_{S}\right)}_{J_{s}}+{\left(\Phi_{S}\right)}_{I}=J_{s}\frac{∆\pi_{s}}{C_{s}}+IE$$

Considering Equation (4) in Equation (8), we obtain the following:

(9) $${\left(\Phi_{S}\right)}_{R}=R_{11}{J_{s}}^{2}+\left(R_{12}+R_{21}\right)J_{s}I+R_{22}I^{2}$$

Taking into account the fact that, in Equation (9), the expressions for $R_{11}$, $R_{12}$ = $R_{21}$ and $R_{22}$ are found in Equation (5), we obtain the following:

(10) $${\left(\Phi_{S}\right)}_{R}=\frac{1}{{C_{s}\omega }_{s}}{J_{s}}^{2}-\frac{2\tau_{s}}{z_{s}F{C_{s}\omega }_{s}}J_{s}I+\frac{{z_{s}}^{2}{C_{s}\omega }_{s}+{\kappa \tau_{s}}^{2}}{\kappa {z_{s}}^{2}{C_{s}\omega }_{s}}I^{2}$$

The internal energy (*U*-energy) in membrane systems can be converted into free energy (*F*-energy) and dissipated energy (*S*-energy) [8]. The fluxes of these parameters satisfy the following equation:

(11) $${\left(\Phi_{U}\right)}_{R}={\left(\Phi_{F}\right)}_{R}+{\left(\Phi_{S}\right)}_{R}$$

where ${\left(\Phi_{U}\right)}_{R}=A^{-1}dU/dt$ is the flux of *U*-energy, ${\left(\Phi_{F}\right)}_{R}=A^{-1}dF/dt$ is the flux of *F*-energy, ${\left(\Phi_{S}\right)}_{R}=TA^{-1}\;d_{i}S/dt$ is the flux of dissipated energy (*S*-energy), $d_{i}S/dt$ is the rate of entropy production in the membrane system caused by irreversible processes (the flux of cumulative entropy production), *T* is the absolute temperature, and *A* is the membrane surface area. Equations (9) and (10) show the R version of the S-energy dissipation. Here, ${\left(\Phi_{S}\right)}_{R}$ is the flux of dissipated energy, i.e., the time change in dissipated energy per unit area of the membrane expressed in W/m^2^. We can calculate ${\left(\Phi_{F}\right)}_{R}$ and ${\left(\Phi_{U}\right)}_{R}$ for the concentration polarisation conditions of the membrane using the following equation:

(12) $${\left(e_{R}\right)}_{max}=\frac{{\left(\Phi_{F}\right)}_{R}}{{\left(\Phi_{U}\right)}_{R}}=\frac{{\left(\Phi_{F}\right)}_{R}}{{\left(\Phi_{F}\right)}_{R}+{\left(\Phi_{S}\right)}_{R}}$$

Transforming Equation (12), we obtain the following:

(13) $${\left(\Phi_{F}\right)}_{R}=\frac{{\left(e_{R}\right)}_{max}}{1-({e_{R})}_{max}}{\left(\Phi_{S}\right)}_{R}$$

(14) $${\left(\Phi_{U}\right)}_{R}=\frac{1}{1-({e_{R})}_{max}}{\left(\Phi_{S}\right)}_{R}$$

where $({e_{R})}_{max}$ is the energy conversion efficiency defined by the means of Kedem–Caplan–Peusner coefficients. Using suitable resistant coefficients of Equation (5) in Equation (7), the energy conversion efficiency coefficient can be presented as follows:

(15) $$({e_{R})}_{max}=\frac{{\tau_{s}}^{2}}{\left({\kappa \tau_{s}}^{2}+{z_{s}}^{2}F^{2}{C_{s}\omega }_{s}\right)+{\left(1+\sqrt{\frac{{z_{s}}^{2}F^{2}{C_{s}\omega }_{s}}{{z_{s}}^{2}F^{2}{C_{s}\omega }_{s}+{\kappa \tau_{s}}^{2}}}\right)}^{2}}$$

From a formal point of view, the cases of ${\left(\Phi_{F}\right)}_{R}$ = 0 and ${\left(\Phi_{U}\right)}_{R}$ = 0 are excluded because in order for the denominators of Equations (13) and (14) to be different from zero, the condition $({e_{R})}_{max}$ ≠ 1 must be satisfied.

The values of $({e_{R})}_{max}$ coefficients are limited by the relations 0 ≤ $({e_{R})}_{max}$ ≤ 1; $({e_{R})}_{max}$ = 0 when $R_{12}R_{21}$ = 0 or $r_{12}r_{21}$ = 0 and $({e_{R})}_{max}$ = 1, when $R_{12}R_{21}$ = $R_{11}R_{22}$ and $r_{12}r_{21}$ = 1. The values of the coefficients $({e_{R})}_{max}$ are limited by the relation 0 ≤ $({e_{R})}_{max}$ ≤ +1. Taking into account Equation (7), in (13), we obtain the following:

(16) $${\left(\Phi_{F}\right)}_{R}=\frac{R_{12}R_{21}}{R_{11}R_{22}{\left(1+\sqrt{1-\frac{R_{12}R_{21}}{R_{11}R_{22}}}\right)}^{2}-R_{12}R_{21}}{\left(\Phi_{S}\right)}_{R}$$

Taking into consideration Equation (7) in Equation (14) and performing the necessary transformations, we obtain the following:

(17) $${\left(\Phi_{U}\right)}_{R}=\frac{R_{11}R_{22}R^{2}}{R_{11}R_{22}R^{2}-R_{12}R_{21}}{\left(\Phi_{S}\right)}_{R}$$

where $R=1+\sqrt{1-\frac{R_{12}R_{21}}{R_{11}R_{22}}}$.

### 2.4. Biomembrane Characteristics

**Figure 4:**
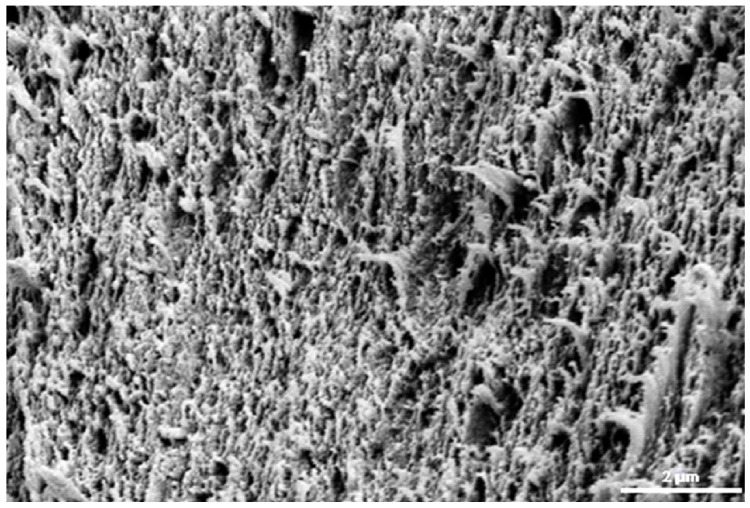
Image of the Ultra Flo 145 Dialyzer membrane obtained from a scanning microscope (Zeiss Supra 35) at 10,000× magnification [8].

Ultra Flo 145 Dialyzer hemodialysis membranes (Artificial Organs Division, Travenol Laboratories S.A., Brussels, Belgium) were used [33]. This membrane is made of regenerated cellulose and is symmetrical, hydrophilic, isotropic and electroneutral. Figure 4 shows the scanning image of the Ultra Flo 145 Dialyser membrane, which has a compact structure with visible cellulose fibre residues. This structure gives the membrane its high stiffness and strength. The Ultra Flo 145 Dialyser membrane was cut in the form of a disc from a haemodialysis hose that was part of a “coiled artificial kidney” used in medicine in the second half of the 20th century.

The membrane separated two Plexiglas vessels of equal volume, filled with aqueous NaCl solutions with different NaCl concentrations. One of the vessels was connected to a calibrated pipette, and the other to a solution reservoir. Ag/AgCl electrodes in the form of a flat disc were placed in each vessel symmetrically on both sides of the membrane. The electrodes had equal thickness and equal surface areas. Electric voltage was applied to the electrodes using a DC power supply. The experiments were performed in an isolated and grounded metal chamber to ensure the elimination of the influence of external electrical interferences and constant temperature (*T* = 295 K).

**Figure 5:**
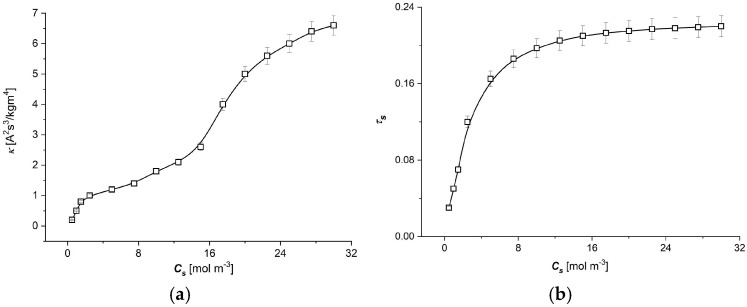
The dependencies of conductivity (κ) (**a**) and transference number ($\tau_{s})$ (**b**) as functions of NaCl concentration in the membrane for the Ultra Flo 145 Dialyzer membrane and aqueous NaCl solutions.

The value of transport coefficients $\omega_{s}$ of the Ultra Flo 145 Dialyser membrane appearing in Equations (2) and (3) in the studied range of NaCl concentrations are constant and equals $\omega_{s}$ = 5.5 × 10^−10^ mol/Ns. In turn, the values of the transport coefficients κ and $\tau_{c}$ in the studied range of NaCl concentrations depend on NaCl concentrations in the membrane. The measured dependencies $\kappa =f(C_{s})$, $\tau_{c}=f(C_{s})$ are presented in Figure 5a,b suitably. The above-mentioned parameters were determined according to the procedure described in this paper [34].

As can be seen from Figure 5, an increase in the average NaCl concentration in the membrane ($C_{s}$) increases both the conductivity coefficient (κ) and the membrane transfer number ($\tau_{s}$). These relationships are nonlinear. In the case of the conductivity coefficient, the initial rapid increase in κ in the low-concentration region changes above 3 mol m^−3^ to a slower increase up to 16 mol m^−3^, after which the rate of increase for the κ coefficient is again higher. The first area of rapid increase in conductivity can be related to the relatively easy passage of ions through the membrane for solutions with low NaCl concentrations. The further slower increase in conductivity can be related to the stronger interaction of ions with the membrane structure, hindering the free flow of ions through the membrane at higher concentrations. If the NaCl concentration is increased further, above 16 mol m^−3^, the high NaCl concentration in the membrane may cause a ‘shielding’ effect of the interaction of the membrane structure with some of the flowing ions, resulting in a greater increase in conductivity with the increasing NaCl concentration in the membrane. A similar effect of the interaction of transported ions across the membrane can be seen in the graph of $\tau_{s}$ as a function of the average concentration in the membrane, the effect being less subtle than conductivity. The increase in the NaCl concentration in the membrane increased the number of ions transporting through the membrane, but with smaller and smaller increments, which may be due to the ions interacting more and more strongly with the membrane during transport as their average concentration in the membrane increases.

## 3. Results and Discussion

### 3.1. The Characteristic $R_{ij}=f(C_{s}$) (i, j ∈ {1, 2})

**Figure 6:**
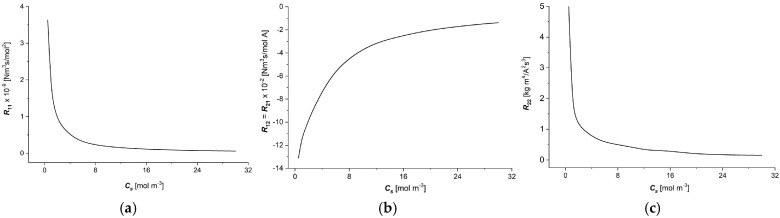
The dependencies $R_{ij}=f(C_{s})$ (i, j ∈ {1, 2}) for aqueous NaCl solutions and the Ultra Flo 145 membrane: (**a**) $R_{11}=f(C_{s})$, (**b**) $R_{12}=R_{21}=f(C_{s})$, and (**c**) $R_{22}=f(C_{s})$.

Calculations of the resistant coefficients $R_{ij}=f(C_{s})$ (i, j ∈ {1, 2}) were performed for the following data: R = 8.31 J/mol K, T = 295 K, F = 9.65 ×10^4^ C/mol, $C_{l}$ = 1 mol/m^3^, and $C_{h}$ ∈ {1 ÷ 20 mol/m^3^}. To calculate the dependencies of resistant coefficients ($R_{ij}$) as functions of NaCl in the membrane, $\left(C_{s}\right)$ (i, j ∈ {1, 2}) and Equation (5) were used. The results of the calculations are presented in Figure 6a–c. From Figure 6a–c, it is clear that the graphs illustrating the dependencies $R_{ij}=f(C_{s})$ are nonlinear functions of $C_{s}$.

The diagonal coefficients of the resistance matrix, which characterise the direct influence of the associated thermodynamic fluxes and forces, decrease nonlinearly with the increasing NaCl concentration in the membrane, which may be related to the easier flow of the solute at higher concentrations. The main changes in diagonal coefficients are observed in low NaCl concentrations, lower than 4 mol m^−3^. In the case of non-diagonal coefficients that are equal, the increase in the average NaCl concentration in the membrane causes a nonlinear decrease in the absolute values of these coefficients. The rate of decrease in absolute values of these coefficients with the increasing NaCl concentration in the membrane being increasingly smaller shows a saturation effect for high NaCl concentrations.

### 3.2. Characteristics $r_{ij}=f(C_{s})$, (i, j ∈ {1, 2, 3})

**Figure 7:**
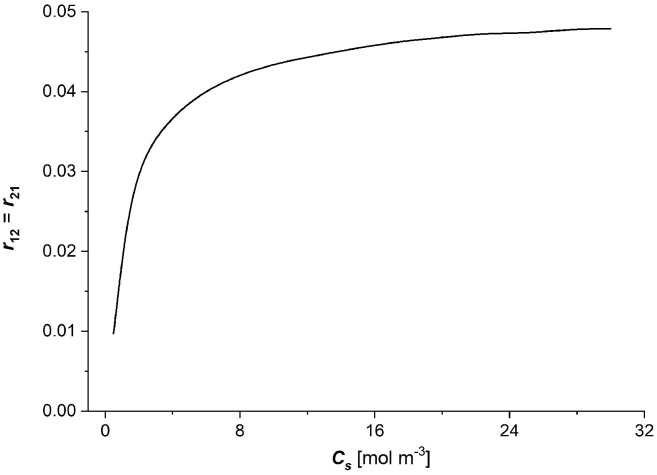
The dependence of the coefficient of degree of coupling between observed processes on NaCl concentration in the Ultra Flo 145 membrane.

Taking into account the dependencies $R_{ij}=f(C_{s})$ (i, j ∈ {1, 2}) shown in Figure 6a–c in Equation (9), the coefficient of degree of coupling between the observed processes ($r_{12}=r_{21}$) as a function of the NaCl concentration in the membrane ($C_{s}$) was calculated and is presented in Figure 7. The curve presented in Figure 7 shows the nonlinear characteristics of this dependence.

As can be seen from Figure 7, the coupling coefficients between membrane processes ($r_{12},r_{21}$) increase with the increasing average NaCl concentration in the membrane. The greatest changes in these coefficients with increasing NaCl concentration in the membrane are observed in the range of small concentrations, up to 8 mol m^−3^. Above this NaCl concentration, a “stabilising” effect of these coupling coefficients can be observed for high values of the NaCl concentration in the membrane.

**Figure 8:**
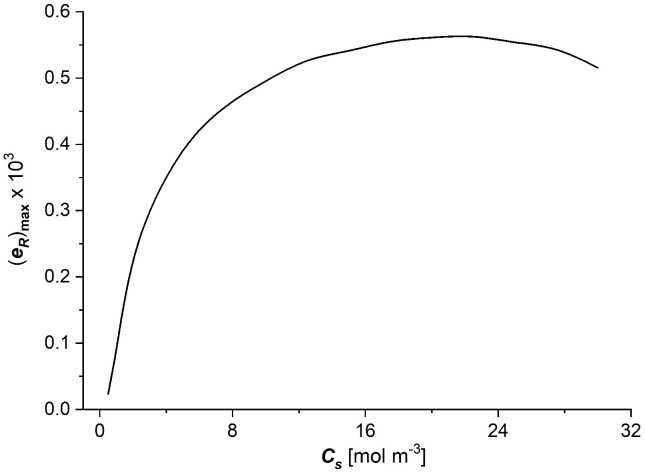
The energy conversion efficiency coefficient ((${e_{R})}_{max})$ as a function of NaCl concentration $(C_{s}$) in the Ultra Flo 145 membrane.

Considering the dependencies $R_{ij}=f(C_{s})$ (i, j ∈ {1, 2}) shown in Figure 6a–c and Equation (7), the dependence of energy conversion efficiency coefficient (${e_{R})}_{max}$ on NaCl concentration in the Ultra Flo 145 membrane was calculated and is presented in Figure 8.

The membrane energy conversion efficiency coefficient ((${e_{R})}_{max})$, increases nonlinearly with the increasing NaCl concentration in the membrane (Figure 8). The highest rate of change in the coefficient is observed for low values of the NaCl concentration in the membrane (up to 8 mol m^−3^). For higher values of NaCl concentrations in the membrane, the value of the coefficient increases slightly and then decreases slightly for very high values of NaCl concentrations in the membrane.

### 3.3. The Characteristics ${\left[{{(\Phi }_{S})}_{R}\right]}_{J_{s}=const}=f(I)$ and ${\left[{{(\Phi }_{F})}_{R}\right]}_{J_{s}=const}=f(I)$

**Figure 9:**
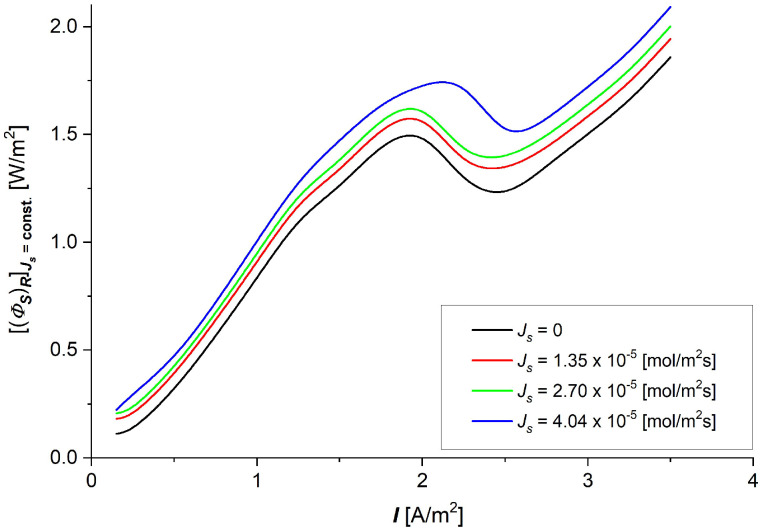
The dissipated energy flux (${{(\Phi }_{S})}_{R})$ as a funtion of current density (*I*) through the Ultra Flo 145 membrane for established NaCl fluxes ($J_{s}=const$).

Taking into account the dependencies $R_{ij}=f(C_{s})$, (i, j ∈ {1, 2}) shown in Figure 6a–c in Equation (9), the dissipated energy flux ${\left((\Phi_{S}\right)}_{R}),$ as a funtion of current density (*I*), with constant NaCl fluxes through the membrane ($J_{s}=\left\{0,\;1.35,\;2.70,\;4.04\right\}\cdot 10^{-5}\;mol/m^{2}s$) was calculated. The results of the calculations are presented in Figure 9. The graphs shown in Figure 9 are nonlinear, showing increasing functions of thermodynamic flux I with local extrema for density currents between 2 and 2.5 A/m^2^.

**Figure 10:**
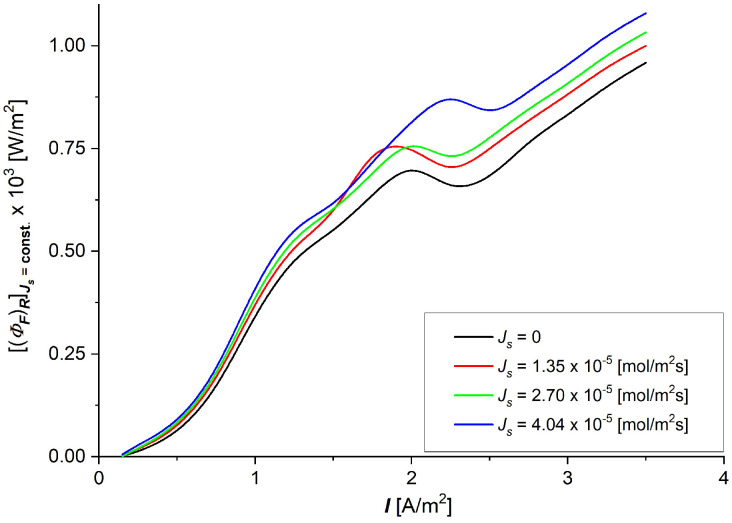
The useful energy flux (${{(\Phi }_{F})}_{R}$) as a funtion of current density (*I*) through the Ultra Flo 145 membrane for established NaCl fluxes ($J_{s}=const$).

The dependencies of the dissipated energy flux during NaCl transport through the UltraFlo 145 membrane in terms of current density presented in Figure 9 show a complex nonlinear nature. In general, it can be stated that the dissipated energy fluxes are relatively small for small values of the current density of NaCl flux through the membrane. The increase in current density through the membrane, with a fixed flux *J*_s_, initially up to a current density of about slightly lower than 2 A/m^2^, causes increases in the dissipated energy flux, which decreases up to 2.5 A/m^2^ and increases with the increasing current density. Taking into account the dependencies (${e_{R})}_{max}=f(C_{s})$ shown in Figure 8 and Equation (9) and the dependencies ${\left[{{(\Phi }_{S})}_{R}\right]}_{J_{s}=const}=f(I)$ presented in Figure 9, the useful energy flux ${\left((\Phi_{F}\right)}_{R})$ as a funtion of current density (*I*) with constant NaCl fluxes throughout the membrane ($J_{s}=\left\{0,\;1.35,\;2.70,\;4.04\right\}\cdot 10^{-5}\;mol/m^{2}s$) was calculated. The results of the calculations are presented in Figure 10.

Taking into consideration Figure 9 and Figure 10, it can be stated that the flux of useful energy ${{(\Phi }_{F})}_{R}$ in membrane processes is much lower than the flux of dissipated energy ${\left(\Phi_{S}\right)}_{R}$ in all ranges of current density and solute fluxes through the membrane (${{(\Phi }_{F})}_{R}\;\approx \;{10^{-3}(\Phi_{S})}_{R}$). For this reason, taking into account Equation (18), it can be concluded that fluxes of dissipated ${{(\Phi }_{S})}_{R}$ and internal ${{(\Phi }_{U})}_{R}$ energies are the same. The fluxes of dissipated energy (${{(\Phi }_{S})}_{R}$) and useful energy (${{(\Phi }_{F})}_{R}$) are important concepts in nonequilibrium thermodynamics. As can be seen from Figure 9 and Figure 10, their dependencies on the current density through the membrane with constant NaCl flux through the membrane are complex nonlinear functions. In both cases, these dependencies show local extrema (maxima and minima). Increasing the current density through the membrane results in mainly an increase in the useful energy flux of membrane processes.

## 4. Discussion

All of the calculated flux–force resistant coefficients ($R_{ij}$) depend nonlinearly on the NaCl concentration in the membrane. The values of diagonal resistive coefficients are positive over the entire range of NaCl concentrations. Non-diagonal resistance coefficients are negative and equal to each other ($R_{12}=R_{21})$ and their absolute values decrease with the increasing NaCl concentration in the Ultra Flo 145 membrane. Nonlinear changes in $R_{ij}$ coefficients cause a more complex force–flux relationship. The greater slope of the characteristics $R_{ij}=f(C_{s})$, (i, j ∈ {1, 2}) causes the effect of a given stimulus on the corresponding flux to be stronger.

From Equation (5), it follows that the positive values of the coefficients $R_{11}$ and $R_{22}$ mean that an increase in the jth stimulus causes an increase in the value of the corresponding *i*-th flux. In the case of nonlinear changes in the coefficients $R_{ij}$, (i, j ∈ {1, 2}), the nature of the characteristics $R_{ij}=f(C_{s})$, (i, j ∈ {1, 2}) is more complex. The nonlinearity of these characteristics is related to the structure of the membrane itself and the frictional interaction between the membrane (*m*), water (*w*) and the ions (with labels 1 and 2) found in the membrane and in the water.

We will outline this problem for electrolyte solutions using the results presented in the work [9]. The starting point is the equations illustrating the frictional interpretation of the coefficients $\omega_{s}$, κ, and $\tau_{s}$.

(18) $$\omega_{s}=\frac{K\;\vartheta }{∆x(f_{sw}+f_{sm})}$$

(19) $$\kappa =\frac{\vartheta \;X\;F^{2}}{f_{1w}^{0}\;∆x}$$

(20) $$\tau_{2}={\left(\frac{C_{s}\varphi_{w}}{X}\right)}^{2}\frac{f_{1w}}{f_{2w}}$$

where *K*—distribution coefficient for salt between aqueous solution and membrane; ∆x—membrane thickness; $f_{sw}$—friction coefficient between *s*-th ion and water molecules; $f_{sm}$—friction coefficient between *s*-th ion and the membrane; *X*—fixed charges concentration in membrane matrix; (indexes: 1—for counterion; 2—for coion); $\varphi_{w}$—volume of water in the membrane; *ϑ*—winding coefficient of channels in the membrane; and $f_{iw}^{0}$—friction coefficient of ion *i* in free solution.

Considering Equations (18)–(20) in Equation (5), we obtain the frictional interpretation of the resistant coefficients as follows:

(21) $$R=\left(\begin{array}{cc} \frac{∆x(f_{sw}+f_{sm})}{C_{s}\;K\;\vartheta } & -\frac{F\;C_{s}{\varphi_{w}}^{2}\;f_{1w}\;\;\left(f_{sw}+f_{sm}\right)}{K\;X\;{\;z}_{s}\;\;f_{2w}f_{1w}^{0}\;\;}\\ -\frac{F\;C_{s}{\varphi_{w}}^{2}\;f_{1w}\;\;\left(f_{sw}+f_{sm}\right)}{K\;X\;{\;z}_{s}\;\;f_{2w}f_{1w}^{0}\;\;} & \frac{∆x}{\vartheta F^{2}}\left\{\frac{f_{1w}^{0}}{X}+{\left[{\left(\frac{C_{s}\varphi_{w}}{X}\right)}^{2}\frac{f_{1w}}{f_{2w}}\right]}^{2}\frac{\left(f_{sw}+f_{sm}\right)}{{z_{s}}^{2}\;C_{s}\;K}\right\} \end{array}\right)$$

As the results from Equation (21) show, the resistance coefficients are connected with the membrane structure and the interactions of the membrane with transported substances. In addition, the interactions between transported substances should be taken into consideration.

## 5. Conclusions

- The moduli of resistant coefficients of the Ultra Flo Dialyser membrane decrease nonlinearly with the increasing NaCl concentration in the membrane, and the main changes in these coefficients are observed for low NaCl concentrations at less than 8 mol/m^3^.
- The coefficients of the degree of coupling of processes and energy conversion efficiency increase nonlinearly with the increasing NaCl concentration in the membrane; the main changes in these coefficients were also observed for low NaCl concentrations, less than 8 mol/m^3^. The value of the energy conversion efficiency coefficients for NaCl concentration in the membrane greater than 8 mol/m^3^ is greater than 0.5.
- The dissipated energy flux during membrane processes is much larger than the useful energy flux for these processes, so the internal energy of the flux is completely converted during membrane processes into the flux of dissipated energy.

## Data Availability

The datasets for this study are available upon request from the corresponding author.

## List of Symbols and Abbreviations

R	gas constant [J/molK]
T	temperature [K]
F	Faraday constant [C/mol]
$\omega_{s}$	solute permeability coefficient [mol/Ns]
$\tau_{c}$	transference number
κ	conductance coefficient
${\Delta \pi }_{s}$	osmotic pressure difference [Pa]
ΔC	solutions concentration difference [mol/m^3^]
$C_{h}$, $C_{l}$	solutes concentrations [mol/m^3^]
$C_{s}$	average concentration of solutes [mol/m^3^]
$J_{s}$	solute flux [mol/m^2^s]
I	electric ionic current [A/m^2^]
$R_{ij}$	the Peusner’s resistant coefficients
$r_{ij}$	coupling coefficient of membrane processes
(${e_{R})}_{max}$	energy conversion efficiency coefficient
${\left(\Phi_{S}\right)}_{R}$	flux of *S*-energy [W/m^2^]
${\left(\Phi_{F}\right)}_{R}$	flux of *F*-energy [W/m^2^]
${{(\Phi }_{U})}_{R}$	flux of *U*-energy [W/m^2^]
*K*	distribution coefficient for salt between aqueous solution and membrane
$\varphi_{w}$	volume of water in membrane
*ϑ*	winding coefficient of channels in membrane
$f_{iw}$	friction coefficient between *i*-th ion and water molecules
$f_{im}$	friction coefficient between *i*-th ion and membrane
$f_{i}^{0w}$	friction coefficient in free solution
PNT	Peusner’s network thermodynamics
NT	network thermodynamics
M	membrane
K-K-P equations	Kedem–Katchalsky–Peusner equations

## References

[1] Baker R. (2012). Membrane Technology and Application.

[2] Radu E.R., Voicu S.I., Thakur V.K. (2023). Polymeric membranes for biomedical applications. Polymers.

[3] Dorotkiewicz-Jach A., Markowitz P., Rachuna J., Arabski M., Drulis-Kawa Z. (2023). The impact of agarose immobilization on the activity of lytic Pseudomonas araginosa phages combined with chemicals. Appl. Microbiol. Biotechnol..

[4] Zhang Y., Yu L., Zhang X.-D., Wang Y.-H., Yang C., Liu X., Wang W.-P., Zhang Y., Li X.-T., Li G. (2023). A smart risk-responding polymer membrane for safer batteries. Sci. Adv..

[5] Bolto B., Zhang J., Wu X., Xie Z. (2020). A review on current development of membranes for oil removal from wastewaters. Membranes.

[6] Yahya L.A., Tobiszewski M., Kubica P., Koronkiewicz S., Vakh C. (2024). Polymeric porous membranes as solid support and protective material in microextraction processes: A review. TrAC Trends Anal. Chem..

[7] Demirel Y. (2007). Nonequilibrium Thermodynamics: Transport and Rate Processes in Physical, Chemical and Biological Systems.

[8] Ślęzak A., Grzegorczyn S.M. (2024). Network derivation of liquid junction potentials in single-membrane system. Membranes.

[9] Katchalsky A., Curran P.F. (1965). Nonequilibrium Thermodynamics in Biophysics.

[10] Paynter H. (1961). Analysis and Design of Engineering Systems.

[11] Meixner J. (1963). Thermodynamics of electrical networks and Onsager-Casimir reciprocal relation. J. Math. Phys..

[12] Peusner L. (1970). The Principles of Network Thermodynamics and Biophysical Applications. Ph.D. Thesis.

[13] Oster G.F., Perelson A., Katchalsky A. (1971). Network Thermodynamics. Nature.

[14] Peusner L. (1983). Hierarchies of irreversible energy conversion systems: A network thermodynamic approach. I. Linear steady state without storage. J. Theor. Biol..

[15] Peusner L. (1985). Hierarchies of irreversible energy conversion systems. II. Network derivation of linear transport equations. J. Theor. Biol..

[16] Peusner L. (1986). Studies in Network Thermodynamics.

[17] Batko K.M., Slezak-Prochazka I., Grzegorczyn S., Ślęzak A. (2014). Membrane transport in concentration polarization conditions: Network thermodynamics model equations. J. Porous Media.

[18] Onsager L. (1931). Reciprocal Relations in Irreversible Processes. I. Phys. Rev..

[19] Peusner L. (1982). Global reaction: Diffusion coupling and reciprocity in linear asymmetric kinetic networks. J. Chem. Phys..

[20] Mikulecky D.C. (1988). When is a mechanism not a mechanism? The network thermodynamic approach to complex systems. Math. Comput. Model..

[21] Mikulecky D.C. (2001). Network thermodynamics and complexity: A transition to relational systems theory. Comput. Chem..

[22] Gawthrop P.J., Pan M. (2022). Network thermodynamics of biological systems: A bond graph approach. Math. Biosci..

[23] Avanzini F., Freitas N., Esposito M. (2023). Circuit theory for chemical reaction networks. Phys. Rev. X.

[24] Zitzmann C., Dächert C., Schmid B., van der Schaar H., van Hemert M., Perelson A.S., van Kuppeveld F.J.M., Bartenschlager R., Binder M., Kaderali L. (2023). Mathematical modeling of plus-strand RNA virus replication to identify broad-spectrum antiviral treatment strategies. PLoS Comput. Biol..

[25] Mogilner A., Oster G. (1996). Cell motility driven by actin polymerization. Biophys. J..

[26] Oster G. (2004). Clocks and patterns in Myxobacteria: A remembrance of Art Winfree. J. Theor. Biol..

[27] Lachenbruch C.A., Diller K.R. (1994). A network thermodynamic model of kidney perfusion. IFAC Proc. Vol..

[28] Horno J., González-Fernández C.F., Hayas A., González-Caballero F. (1989). Simulation of concentration polarization in electrokinetic processes by network thermodynamic methods. Biophys. J..

[29] Mamedov M.M. (2003). Phenomenological derivation of the Onsager reciprocal relations. Tech. Phys. Lett..

[30] Filippov A.N. (2022). A Cell model of an ion-exchange membrane. capillary-osmosis and reverse-osmosis coefficients. Colloid J..

[31] Kedem O., Caplan S.R. (1965). Degree of coupling and its relation to efficiency of energy conversion. Trans. Faraday Soc..

[32] Caplan S.R. (1971). Nonequilibrium thermodynamics and its application to bioenergetics. Curr. Top. Bioenerg..

[33] Twardowski Z.J. (2008). History of hemodialyzers’ designs. Hemodial. Int..

[34] Grzegorczyn S. (2006). Effects of Concentration Polarization of Flat Bacterial Cellulose Membranes.

